# Obtaining P3P Privacy Policies for Composite Services

**DOI:** 10.1155/2014/961659

**Published:** 2014-07-13

**Authors:** Yi Sun, Zhiqiu Huang, Changbo Ke

**Affiliations:** College of Computer Science and Technology, Nanjing University of Aeronautics and Astronautics, Nanjing, Jiangsu 210016, China

## Abstract

With the development of web services technology, web services have changed from single to composite services. Privacy protection in composite services is becoming an important issue. P3P (platform for privacy preferences) is a privacy policy language which was designed for single web services. It enables service providers to express how they will deal with the privacy information of service consumers. In order to solve the problem that P3P cannot be applied to composite services directly, we propose a method to obtain P3P privacy policies for composite services. In this method, we present the definitions of *Purpose*, *Recipient*, and *Retention* elements as well as *Optional* and *Required* attributes for P3P policies of composite services. We also provide an instantiation to illustrate the feasibility of the method.

## 1. Introduction

Nowadays Internet has become one of the major ways for people to get services. More and more people are accustomed to using web services. And with the development of web services technology, web services have changed from single to composite services. Web service composition is an approach to build new composite services by combining existing services. It not only reuses existing web services to improve the efficiency of service development, but also satisfies service consumers' multifunctional demands. However, whether services are single or composite, service consumers' privacy information is inevitably collected by service providers. And it is hard for service consumers to control the disclosure of their privacy information. In order to prevent the privacy information from being misused, service providers are requested to publish their privacy policies. Based on the privacy policies published by service providers, service consumers are able to know what service providers will do with their privacy information.

P3P (platform for privacy preferences) [1] was released by World Wide Web Consortium (W3C) in April 2002. It provides a standard and machine-understandable privacy policy. W3C also designs APPEL (A P3P Preference Exchange Language) [2] which allows service consumers to specify their privacy preferences. A P3P user agent can compare the P3P policies of service providers with the privacy preferences of service consumers. The comparison results enable the service consumers to decide whether to use the services or not. Since P3P was originally designed for single services, it cannot be applied for composite services directly. A composite service may consist of several independent web services which are called member services. All these member services have their own P3P policies which may specify different privacy practices of the same private data. How to isolate these discrepancies is one challenge. How to obtain the P3P policy for a composite service consisting of several services is another challenge.

In this paper, we firstly present the definitions of* Purpose*,* Recipient*, and* Retention* elements as well as* Optional* and* Required* attributes for P3P policies of composite services. Secondly, based on these definitions, we obtain P3P privacy policies for composite services. Finally, we provide an instantiation to illustrate how to obtain a P3P privacy policy of a composite service concretely.

The rest of this paper is organized as follows.  Section 2 describes the syntax of P3P privacy policy.  Section 3 proposes a method to obtain P3P privacy policies for composite services by defining the* Purpose*,* Recipient*, and* Retention* elements as well as the* Optional* and* Required* attributes for P3P policies of composite services. A case study is presented to prove the feasibility of the method in Section 4. Related work is discussed in Section 5.  Section 6 concludes the paper.

## 2. P3P Syntax

P3P privacy policies inform service consumers how service providers will deal with their privacy information. The overall structure of a P3P policy is shown in Algorithm 1. A P3P privacy policy is described by* Policy* consisting of an* Entity* element, an* Access* element, a* Disputes-Group* element, zero or more* Extension* elements, and one or more* Statement* elements.* Entity* gives a precise description of the legal entity making the representation of the privacy practices.* Access* indicates whether the site provides access to various kinds of information.* Dispute-Group* describes dispute resolution procedures that may be followed for disputes about a service's privacy practices.

*Statement* is the core of P3P privacy policy. It describes how a website collects and uses the private data of service consumers.* Statement* comprises* Purpose*,* Recipient*,* Retention*, and* Data-Group* elements.* Purpose* states for what purpose the private data of service consumers may be used. It has six predefined values such as* current*,* admin*, and* develop*.* Recipient* describes to whom the private data of service consumers will be exposed.* Retention* states how long the private data of service consumers will be retained by service providers.* Data-Group* contains a list of private data (*Data* element) of service consumers which may be collected by service providers and data categories (*Categories* element). Moreover,* Data*,* Purpose*, and* Recipient* are either optional or mandatory by taking an optional attribute called* Optional* for the former and* Required* for the latter two. The value of* Optional* is either* no* (default value) when the data must be collected or* yes* when the data is optional. The value of* Required* can be* always* (default value),* opt-out*, or* opt-in*.* Always* means the purpose/recipient is always needed.* Opt-out* means the data may be used for the purpose/may be distributed to the recipient unless the user requests that it not be used in this way.* Opt-in* means the data may be used for the purpose/may be distributed to the recipient only when the user affirmatively requests this use.  Algorithm 2 shows an example P3P policy from http://www.walmart.com/ [3].

## 3. P3P Policies in Composite Services

Web service composition uses web services, no matter single or composite, as fundamental elements to create new services. It not only reuses existing services but also improves the efficiency of service development. The application of service composition is supported by many techniques, such as BPEL [4] and WSDL [5]. BPEL specifies the internal business process of a composite service. WSDL describes the interfaces of member services. Through these interfaces, member services can be invoked. At present there are many existing approaches to service composition, some of which are abstract methods and some of which aim to be industry standards [6–9].

P3P is one of the structured privacy policy languages widely used in the world today [10, 11]. It specifies how service providers will deal with the privacy information of service consumers. However, P3P cannot be applied for composite services directly because it was originally designed for single services. There is a need to research on P3P privacy policies for composite services.* Statement* element states the way service providers will handle the privacy information of service consumers, which is a top concern to service consumers. Therefore, the main emphasis of our research on P3P privacy policies for composite services is the* Statement* element. And in a* Statement*, the major elements are* Data*,* Purpose*,* Recipient*, and* Retention*.

As a composite service consists of several member services, the service providers of these member services have their own P3P policies that may specify different privacy practices of the same private data. For example, a composite service called Service A is constituted of three single services, and the P3P privacy policies of these services are called Policy_1_, Policy_2_, and Policy_3_ as shown in Algorithms 3, 4, and 5. From Algorithms 3, 4, and 5 the* Retention* value of #user.name in Policy_1_ is* indefinitely* which means the data is retained for an indeterminate period of time while the* Retention* value of #user.name in Policy_2_ is* stated-purpose* which means the data is retained to meet the stated purpose. The* Purpose* value of #user.name in Policy_1_ is* current* while the* Purpose* value of #user.name in Policy_2_ is* current* and* telemarketing*. The* Recipient* value of #user.home-info in Policy_2_ is* ours* and* unrelated* while the* Recipient* value of #user.home-info in Policy_3_ is* ours* and* same*. In addition, the* Optional* value of #user.name in Policy_1_ is* no* which means the data must be collected while the* Optional* value of #user.name in Policy_2_ is* yes* which means the data is optional. Then what are the* Purpose*,* Recipient*, and* Retention* values of #user.name and #user.home-info in the P3P policy of Service A? What are the* Optional* values of #user.name and #user.home-info? In this connection, we define the values of* Purpose*,* Recipient*, and* Retention* elements as well as the values of* Optional* and* Required* attributes for the P3P privacy policies of composite services. Based on these definitions, we can obtain P3P privacy policies for composite services. It should be noted that the semantic associations of* Purpose*,* Recipient*,* Retention*, and* Data* in* Statement* are not changed all the time. That is to say, the corresponding relationships among* Purpose*,* Recipient*,* Retention*, and* Data* keep the same.

### 3.1. *Purpose* Definition

In* Statement*,* Purpose* indicates the intended use of privacy information. It has twelve predefined values such as* current*,* admin*, and* develop*. When several services are combined into a composite service, their functions have not changed. These services are just reused to form a more powerful service. So when a web service is turned from an independent service to a member service, the* Purpose* values in its privacy policy do not change. We define* Purpose* values of a* Data* element in privacy policy of composite service by the union of* Purpose* values of the* Data* element in privacy policies of member services. And if a* Data* element is not included in the privacy policy of a member service, we set the* Purpose *values of the* Data* element in the service's privacy policy to be an empty set.

**Definition 1 .**

A composite service consists of *n* member services. The sets of* Purpose* values of a* Data* element in privacy policies of member services are denoted by *P*, noted as *P*_*S*_1__, *P*_*S*_2__,…, *P*_*S*_*n*__. The set of* Purpose* values of the* Data* element in privacy policy of composite service is denoted by *P*_CS_, *P*_CS_ = *P*_*S*_1__ ∪ *P*_*S*_2__ ∪ ⋯∪*P*_*S*_*n*__.

According to Definition 1, we can get the sets of* Purpose* values of* Data* elements in privacy policy of Service A:

(1)  PA_user.name=P1_user.name∪P2_user.name∪P3_user.name={current}∪{current,telemarketing}∪∅={current,telemarketing}, PA_user.home-info=P1_user.home-info  ∪P2_user.home-info∪P3_user.home-info=∅∪{current,telemarketing}∪{telemarketing}={current,telemarketing}.

### 3.2. *Recipient* Definition

In a P3P privacy policy,* Recipient* element has six predefined values which are* ours*,* delivery*,* same*,* other-recipient*,* unrelated*, and* public*.* Ours* refers to the service provider and/or a third party that processes data only on behalf of the service provider for the completion of the stated purposes.* Delivery* refers to legal entities performing delivery services that may use data for purposes other than completion of the stated purpose.* Same* represents legal entities that use the data on their own behalf under equable practices.* Other-recipient* represents legal entities that are constrained by and accountable to the original service provider but may use the data in a way not specified in the service provider's practices.* Unrelated* represents legal entities whose data usage practices are not known by the original service provider.* Public* refers to public fora. In addition to* ours* and* public*, the other four predefined values represent a set of recipients, respectively. These recipients have been known explicitly by service providers. Therefore,* delivery*,* same*,* other-recipient*, and* unrelated* denote, respectively, the set of delivery services possibly following different practices, the set of legal entities following equable practices, the set of legal entities following different practices, and the set of legal entities whose data usage practices are not known. We define the six predefined values of* Recipient* element in privacy policies of composite services as follows.

**Definition 2 .**

A composite service consists of *n* member services. If* Recipient* values of a* Data* element in privacy policies of member services include* ours*, the* Recipient* values of the* Data* element in privacy policy of composite service include* ours* as well.

**Definition 3 .**

A composite service consists of *n* member services. The sets of* delivery* values of a* Data* element in privacy policies of member services are noted as delivery_*S*_1__ , delivery_*S*_2__,…, delivery_*S*_*n*__. The set of* delivery* values of the* Data* element in privacy policy of composite service is denoted as delivery_CS_, delivery_CS_ = delivery_*S*_1__ ∪ delivery_*S*_2__ ∪ ⋯∪delivery_*S*_*n*__.

If a* Data* element is not included in privacy policy of a member service or* Recipient* values of the* Data* element in privacy policy of a member service do not include* delivery*, the set of* delivery* values in the service's privacy policy is set to be an empty set.

**Definition 4 .**

A composite service consists of *n* member services. The sets of* same* values of a* Data* element in privacy policies of member services are noted as same_*S*_1__, same_*S*_2__,…, same_*S*_*n*__. The set of* same* values of the* Data* element in privacy policy of composite service is denoted as same_CS_, same_CS_ = same_*S*_1__ ∪ same_*S*_2__ ∪ ⋯∪same_*S*_*n*__.

If a* Data* element is not included in privacy policy of a member service or* Recipient* values of the* Data* element in privacy policy of a member service do not include* same*, the set of* same* values in the service's privacy policy is set to be an empty set.

**Definition 5 .**

A composite service consists of *n* member services. The sets of* other-recipient* values of a* Data* element in privacy policies of member services are noted as other-recipient_*S*_1__, other-recipient_*S*_2__,…, other-recipient_*S*_*n*__. The set of* other-recipient* values of the* Data* element in privacy policy of composite service is denoted as other-recipient_CS_, other-recipient_CS_ = other-recipient_*S*_1__ ∪ other-recipient_*S*_2__ ∪ ⋯∪other-recipient_*S*_*n*__.

If a* Data* element is not included in privacy policy of a member service or* Recipient* values of the* Data* element in privacy policy of a member service do not include* other-recipient*, the set of* other-recipient* values in the service's privacy policy is set to be an empty set.

**Definition 6 .**

A composite service consists of *n* member services. The sets of* unrelated* values of a* Data* element in privacy policies of member services are noted as unrelated_*S*_1__, unrelated_*S*_2__,…, unrelated_*S*_*n*__. The set of* unrelated* values of the* Data* element in privacy policy of composite service is denoted as unrelated_CS_, unrelated_CS_ = unrelated_*S*_1__ ∪ unrelated_*S*_2__ ∪ ⋯∪unrelated_*S*_*n*__.

If a* Data* element is not included in privacy policy of a member service or* Recipient* values of the* Data* element in privacy policy of a member service do not include* unrelated*, the set of* unrelated* values in the service's privacy policy is set to be an empty set.

**Definition 7 .**

A composite service consists of *n* member services. If* Recipient* values of a* Data* element in privacy policies of member services include* public*, the* Recipient* value of the* Data* element in privacy policy for composite service is* public*. And same_CS_ = other-recipient_CS_ = unrelated_CS_ = delivery_CS_ = *∅* will be set.

According to Definitions 2~7, we can get* Recipient* values of* Data* elements in privacy policy of Service A as shown in Table 1. The* Recipient* value of #user.name in privacy policy of Service A is ours. The* Recipient *values of #user.home-info in privacy policy of Service A are ours, same_3_user.home-info_, and unrelated_2_user.home-info_.

### 3.3. *Retention* Definition

In P3P privacy policies,* Retention* element has five predefined values which are* no-retention*,* stated-purpose*,* legal-requirement*,* business-practices*, and* indefinitely*.* No-retention* means information is not retained for more than a brief period of time necessary to make use of it during the course of a single online interaction.* Stated-purpose* means information is retained to meet the stated purpose.* Legal-requirement* means information is retained to meet a stated purpose, but the retention period is longer because of a legal requirement or liability.* Business-practices* indicates information is retained under a service provider's stated business practices.* Indefinitely* indicates information is retained for an indeterminate period of time. When the* Retention* value is* stated-purpose*,* legal-requirement*, or* business-practices*, service providers will give the specific destruction time. If the* Retention* value is* no-retention*, we set the retention time to be zero noted as 0. And if the* Retention* value is* indefinitely*, we set the retention time to be infinity noted as  *∞*. We choose the longest retention time of a* Data* element in the privacy policies of member services as the retention time of the* Data* element in privacy policy of composite service. Moreover, we use specific time instead of* stated-purpose*,* legal-requirement*, and* business-practices* to represent* Retention* values in the privacy policies of composite services. And if a* Data* element is not included in privacy policy of a member service, we set the* Retention *value of the* Data* element in the service's privacy policy to be null.

**Definition 8 .**

A composite service consists of *n* member services. The specific time corresponding to the* Retention* values of a* Data* element in privacy policies of member services is denoted by *T*, noted as *T*_*S*_1__, *T*_*S*_2__,…, *T*_*S*_*n*__. The* Retention *value of the* Data* element in privacy policy of composite service is denoted by *T*_CS_. If 0 < max⁡{*T*_*S*_1__, *T*_*S*_2__,…, *T*_*S*_*n*__} < *∞*, *T*_CS_ = max⁡{*T*_*S*_1__, *T*_*S*_2__,…, *T*_*S*_*n*__}. If max⁡{*T*_*S*_1__, *T*_*S*_2__,…, *T*_*S*_*n*__} = 0, *T*_CS_ = no-retention. If max⁡{*T*_*S*_1__, *T*_*S*_2__,…, *T*_*S*_*n*__} = *∞*, *T*_CS_ = indefinitely.

The* Retention *values of #user.name and #user.home-info in privacy policies of member services are showed in Table 2. According to Definition 8, we can get* Retention* values of* Data* elements in privacy policy of Service A as follows:

(2) TA_user.name =max⁡{T1_user.name,T2_user.name,T3_user.name} =∞=indefinitely,TA_user.home-info =max⁡{T1_user.home-info,T2_user.home-info,T3_user.home-info} =∞=indefinitely.

### 3.4. Attributes Definition

In a P3P privacy policy,* Data*,* Purpose,* and* Recipient *elements can take an optional attribute called* Optional* for the former and* Required* for the latter two. The* Optional* value is either* no* when the data is needed or* yes* when the data is optional. And if the* Optional* value of data is not explicitly specified, the* Optional* value of the data will take the default value (*no*). The* Required *value for* Purpose* elements can be* always, opt-in,* or* opt-out. Always* means the purpose is always required.* Opt-in* means data may be used for the purpose only when the user affirmatively requests this use.* Opt-out* means data may be used for the purpose unless the user requests that it not be used in this way. And if the* Required* value of a purpose is not explicitly specified, the* Required* value of the purpose will take the default value (*Always*). The* Required *value for* Recipient* elements with the exception of* ours* can be* always, opt-in,* or* opt-out. Always* means the recipient is always required.* Opt-in* means data may be distributed to the recipient only when the user affirmatively requests this use.* Opt-out* means data may be distributed to the recipient unless the user requests that it not be used in this way. And if the* Required* value of a recipient is not explicitly specified, the* Required* value of the recipient will take the default value (*Always*). We define the values of* Optional* and* Required* attributes in privacy policies of composite services as follows.

**Definition 9 .**

A composite service consists of *n* member services. The* Optional* values of a* Data *element in privacy policies of member services are denoted by *O*, noted as *O*_*S*_1__, *O*_*S*_2__,…, *O*_*S*_*n*__. The* Optional *value of the* Data* element in privacy policy of composite service is denoted by *O*_CS_. If one of *O*_*S*_1__, *O*_*S*_2__,…, *O*_*S*_*n*__ is* no*, then *O*_CS_ is* no*; otherwise *O*_CS_ is* yes*.

If a* Data* element is not included in privacy policy of a member service, the* Optional* value of the* Data* element in the service's privacy policy is set to be null.

**Definition 10 .**

A composite service consists of *n* member services. The* Required* values of a* Purpose* value in privacy policies of member services are denoted by Req, noted as Req_*S*_1__, Req_*S*_2__,…, Req_*S*_*n*__. The* Required *value of the* Purpose* value in privacy policy of composite service is denoted by Req_CS_. If one of Req_*S*_1__, Req_*S*_2__,…, Req_*S*_*n*__ is* always*, then Req_CS_ is* always*. If all of Req_*S*_1__, Req_*S*_2__,…, Req_*S*_*n*__ are* opt-in*, then Req_CS_ is* opt-in.* Otherwise Req_CS_ is* opt-out*.

If a* Purpose* value is not included in the privacy policy of a member service, the* Optional* value of the* Purpose* value in the service's privacy policy is set to be null.

**Definition 11 .**

A composite service consists of *n* member services. If a* Recipient* value is not* ours* or* public*, the* Required* value of the* Recipient *value in privacy policy of composite service is the same as the value in privacy policies of member services.

If a* Recipient *value is not included in privacy policies of member services, the* Required *value of the* Recipient* value in privacy policy of composite service is set to be null.

**Definition 12 .**

A composite service consists of *n* member services. The* Required* value of* public* in privacy policies of member services is denoted by Red, noted as Red_*S*_1__, Red_*S*_2__,…, Red_*S*_*n*__. The* Required *value of* public* in privacy policy of composite service is denoted by Red_CS_. If one of Red_*S*_1__, Red_*S*_2__,…, Red_*S*_*n*__ is* always*, then Red_CS_ is* always*. If all of Red_*S*_1__, Red_*S*_2__,…, Red_*S*_*n*__ are* opt-in*, then Red_CS_ is* opt-in*. Otherwise Red_CS_ is* opt-out*.

If* public* is not included in privacy policy of a member service, the* Required* value of* public* in the service's privacy policy is set to be null.

According to Definition 9, we can get* Optional* values of* Data* elements in privacy policy of Service A as shown in Table 3. The* Optional* value of #user.name in privacy policy of Service A is no. The* Optional* value of #user.home-info in privacy policy of Service A is no.

According to Definition 10, we can get* Required* values of* Purpose* values in privacy policy of Service A as shown in Table 4. The* Required* values of* Purpose* values in privacy policy of Service A are all always.

According to Definitions 11~12, we can get* Required* values of* Recipient* values in privacy policy of Service A as shown in Table 5. The* Required *value of same_3_user.home-info_ in privacy policy of Service A is always. The* Required *value of unrelated_2_user.home-info_ in privacy policy of Service A is opt-out.

By Definitions 1~12, we have got the* Purpose* values,* Recipient* values,* Retention* values, and* Optional* values of* Data* elements in Service A as well as the* Required* values of* Purpose* and* Recipient* elements. On the basis of the semantic associations of* Purpose*,* Recipient*,* Retention,* and* Data *elements, we can obtain the P3P privacy policy for Service A as shown in Algorithm 6.

## 4. Case Study

Consider an online travel broker service named TravelBroker. The TravelBroker service consists of FlightBooking, HotelReservation, and Payment services. People can book plane tickets, reserve hotel rooms, and pay online through TravelBroker. The P3P privacy policies of FlightBooking service, HotelReservation service, and Payment service are called Policy_1_, Policy_2_, and Policy_3_, respectively, as shown in Algorithms 7, 8, and 9. Next we will show how to obtain P3P privacy policy for TravelBroker.

According to Definition 1, we can get the sets of* Purpose* values of* Data* elements in privacy policy of TravelBroker:

(3) PCS_user.IDCardNo=P1_user.IDCardNo∪P2_user.IDCardNo ∪P3_user.IDCardNo={current}∪{current}∪∅={current},PCS_user.Name=P1_user.Name∪P2_user.Name ∪P3_user.Name={current,contact}∪{current,contact} ∪{current}={current,contact},PCS_user.Mobile=P1_user.Mobile∪P2_user.Mobile ∪P3_user.Mobile={current,contact}∪{current,contact} ∪{current}={current,contact},PCS_user.BankCardNo=P1_user.BankCardNo∪P2_user.BankCardNo ∪P3_user.BankCardNo=∅∪∅∪{current}={current}.

According to Definitions 2~7, we can get* Recipient* values of* Data* elements in privacy policy of TravelBroker as shown in Table 6. The* Recipient* value of #user.IDCardNo in privacy policy of TravelBroker is ours. The* Recipient* values of #user. Name in privacy policy of TravelBroker are ours, same_1_user.Name_, and unrelated_2_user.Name_. The* Recipient* values of #user.Mobile in privacy policy of TravelBroker are ours, same_1_user.Mobile_, and unrelated_2_user.Mobile_. The* Recipient* value of #user.BankCardNo in privacy policy of TravelBroker is ours.

Table 7 shows the* Retention* values of #user.IDCardNo, #user.Name, #user.Mobile, and #user.BankCardNo in privacy policies of member services. According to Definition 8, we can get* Retention* values of* Date* elements in privacy policy of TravelBroker as follows:

(4) TCS_user.IDCardNo =max⁡{T1_user.IDCardNo,T2_user.IDCardNo,T3_user.IDCardNo} =1  month,TCS_user.Name =max⁡{T1_user.Name,T2_user.Name,T3_user.Name} =∞=indefinitely,TCS_user.Mobile =max⁡{T1_user.Mobile,T2_user.Mobile,T3_user.Mobile} =∞=indefinitely,TCS_user.BankCardNo =max⁡{T1_user.BankCardNo,T2_user.BankCardNo,T3_user.BankCardNo} =1  month.

According to Definition 9, we can get* Optional* values of* Data* elements in privacy policy of TravelBroker as shown in Table 8. The* Optional* value of #user.IDCardNo in privacy policy of TravelBroker is no. The* Optional* value of #user.Name in privacy policy of TravelBroker is no. The* Optional* value of #user.Mobile in privacy policy of TravelBroker is no. The* Optional* value of #user.BankCardNo in privacy policy of TravelBroker is no.

According to Definition 10, we can get* Required* values of* Purpose* values in privacy policy of TravelBroker as shown in Table 9. The* Required* value of current in privacy policy of TravelBroker is always. The* Required* value of contact in privacy policy of TravelBroker is opt-out.

According to Definitions 11~12, we can get* Required* values of* Recipient* values in privacy policy of TravelBroker as shown in Table 10. The* Required *value of same_1_user.Name_ in privacy policy of TravelBroker is always. The* Required *value of unrelated_2_user.Name_ in privacy policy of TravelBroker is opt-out. The* Required *value of same_1_user.Mobile_ in privacy policy of TravelBroker is always. The* Required *value of unrelated_2_user.Mobile_ in privacy policy of TravelBroker is opt-out.

By Definitions 1~12, we have got the* Purpose* values,* Recipient* values,* Retention* values, and* Optional* values of* Data* elements in TravelBroker as well as the* Required* values of* Purpose* and* Recipient* element. On the basis of the semantic associations of* Purpose*,* Recipient*,* Retention,* and* Data *elements, we can obtain the P3P privacy policy for TravelBroker as shown in Algorithm 10.

## 5. Related Work

When people enjoy the convenient, efficient, and flexible services on the Internet, their privacy information is inevitably collected by service providers. References [12, 13] assessed the risks of privacy abuse by game theory. The conclusion was that service providers tended to seek for undue interests by misusing and exposing users' privacy information. In order to prevent users' privacy information from being abused, service providers are asked to publish their privacy policies on their websites. P3P privacy policy has been used by more and more websites. By July 2003, 30% of the top 100 websites had used P3P. And 23% of the top 500 websites had used P3P [14].

Some scholars have researched on the semantics for P3P privacy policy and the relationships among several P3P privacy policies. The work from Hogben expressed P3P privacy policy formally by an OWL ontology [15]. This work had been written to the W3C Working Group Note. Yu et al. proposed data-centric formal semantics for P3P policies, which precisely and intuitively modeled the relationships between different components of P3P statements [16]. Agrawal et al. enunciated key privacy principles for privacy-aware database systems and proposed a strawman design for the database systems using purpose-centric base [17]. Boontawee Suntisrivaraporn and Khurat proposed semantics for P3P employing a data-purpose centric relational table [18]. They used an OWL ontology to systematically and precisely describe P3P privacy policy. In our previous work, we put forward data-recipient centric formal semantics for P3P policies, which supported the semantic conflict detection of P3P [19]. May et al. proposed two flexible policy relations derived from bisimulation in process calculi [20]. They illustrated the relations using examples from P3P. Nikolaos Papanikolaou et al. presented an approach to check for refinement between policies [21]. They automatically generated CSP models from P3P policies and then performed various tests using the FDR model checker.

With the wide application of web service composition, the protection of users' privacy information in composite services attracts more and more attention. However, there are only a few scholars studying the application of P3P privacy policies in web service composition. Khurat et al. enhanced P3P to be able to support composite services, proposed a formal semantic for P3P employing a data-purpose centric relational table, and defined combining methods to obtain privacy policies of composite services [22]. However, due to the syntax of P3P being extended, P3P privacy policies of composite services generated by such methods could not be directly used to match with users' privacy preferences. Our proposed method does not change the syntax of P3P. P3P privacy policies of composite services obtained by our method can match with users' privacy preferences directly. Michele Chinosi and Trombetta checked whether a BPeX-represented business process was compliant with a P3P privacy policy by introducing a data model for BPMN and a corresponding XML-based representation called BPeX [23]. Li et al. proposed a graph-transformation based framework to check whether an internal business process adhered to the organizations' P3P privacy policies [24]. Both [23, 24] applied P3P privacy policies in web service composition by business processes. However, they did not propose methods to obtain P3P privacy policies for composite services, yet assuming that P3P privacy policies of composite services had already existed. Our work proposes a method to obtain P3P privacy policies for composite services, which is the foundation of their work. Dong et al. presented an approach to implement privacy policy aggregation with P3P [10]. But they did not consider the internal relationship between* Data* elements and* Purpose*,* Recipient*, and* Retention* elements. In our work, we generate the* Purpose*,* Recipient*, and* Retention* values for each* Data* element, respectively, which avoids potential conflicts between* Data *elements and other elements.

## 6. Conclusions and Future Work 

Privacy protection in composite services has become an important issue. P3P is an existing technology employed to protect privacy. In order to apply P3P directly to composite services, we propose a method to obtain P3P privacy policies for composite services in the paper. We present the definitions of* Purpose*,* Recipient*, and* Retention* elements as well as* Optional* and* Required* attributes for P3P policies of composite services in the method and provide an instantiation to demonstrate the feasibility of the method.

The base data schema of P3P is defined in a hierarchy. It will cause conflicts of data hierarchy constraints if the upper level data has more strict constraints than its lower level data. For example, #user.bdate is the higher level data relative to #user.bdate.ymd.year. If the* Optional* value of #user.bdate is* yes*, the* Optional* value of #user.bdate.ymd.year can be* no* or* yes*. But if the* Optional* value of #user.bdate is* no*, the* Optional* value of #user.bdate.ymd.year must be* no*. In this paper, there is no conflict of data hierarchy constraints in P3P privacy policies. So we do not consider the conflicts of data hierarchy constraints when obtaining P3P privacy policies for composite services. As future work, we plan to consider the conflicts of data hierarchy constraints and enhance our method to resolve it.

## Figures and Tables

**Algorithm 1:**
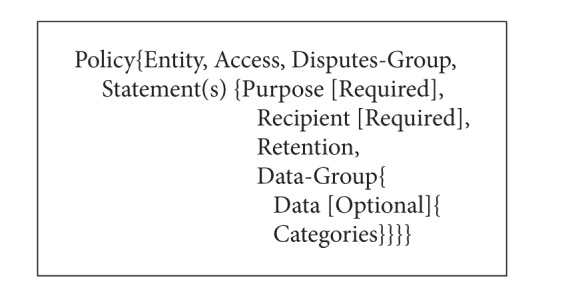
The P3P policy structure and an example P3P policy from http://www.walmart.com/ (the structure of P3P privacy policy).

**Algorithm 2:**
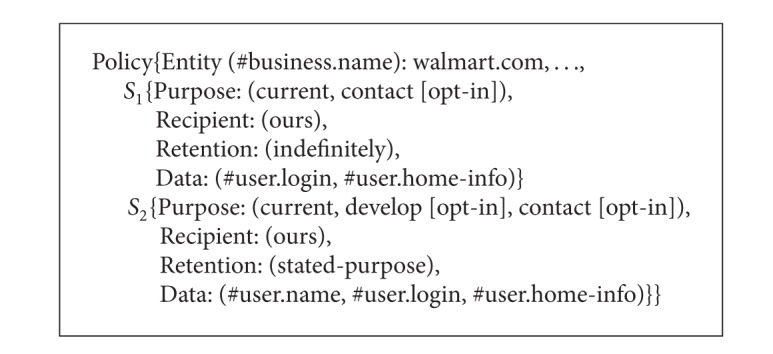
The P3P policy structure and an example P3P policy from http://www.walmart.com/ (a P3P policy from walmart.com).

**Algorithm 3:**
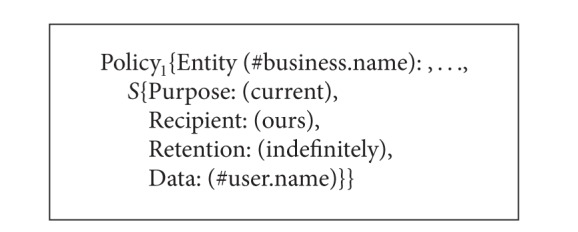
P3P privacy policies of three single services (Policy_1_).

**Algorithm 4:**
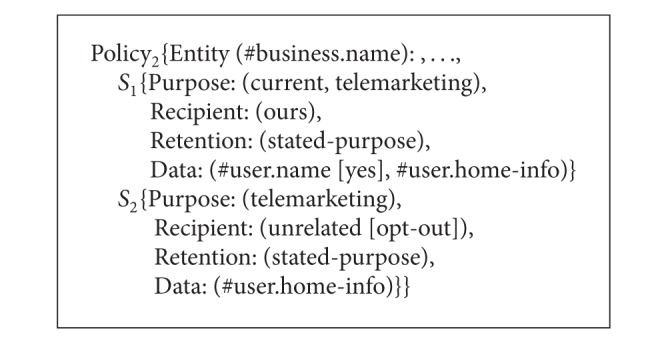
P3P privacy policies of three single services (Policy_2_).

**Algorithm 5:**
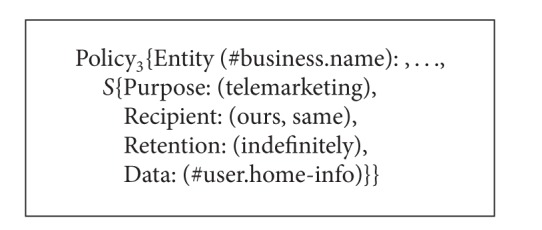
P3P privacy policies of three single services (Policy_3_).

**Algorithm 6:**
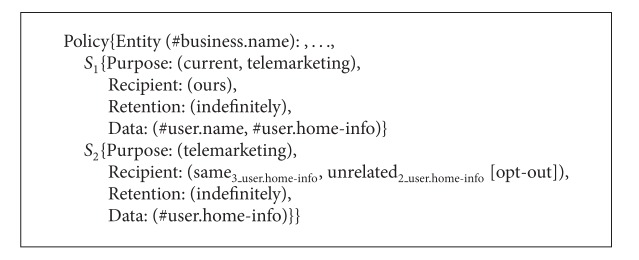
P3P privacy policy for Service A.

**Algorithm 7:**
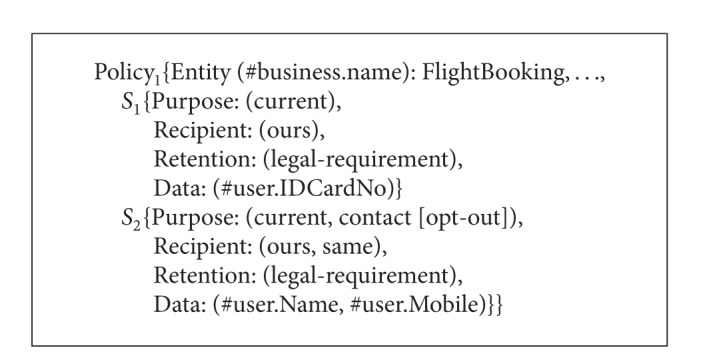
P3P policies of FlightBooking service, HotelReservation service, and Payment service (P3P policy of FlightBooking service).

**Algorithm 8:**
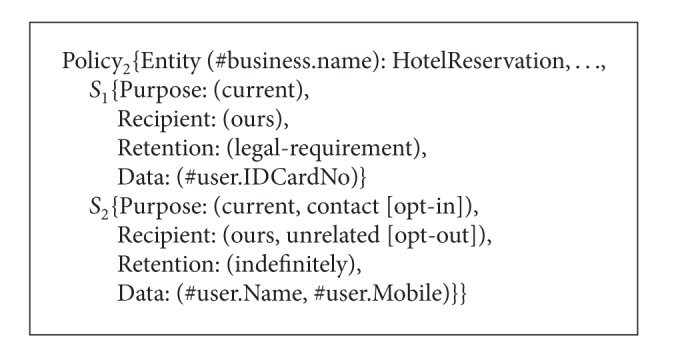
P3P policies of FlightBooking service, HotelReservation service, and Payment service (P3P policy of HotelReservation service).

**Algorithm 9:**
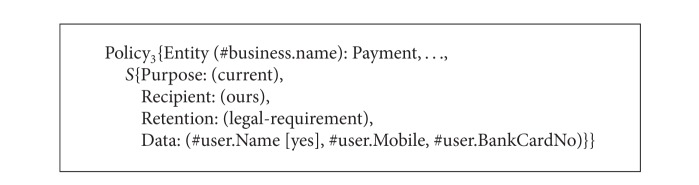
P3P policies of FlightBooking service, HotelReservation service, and Payment service (P3P policy of Payment service).

**Algorithm 10:**
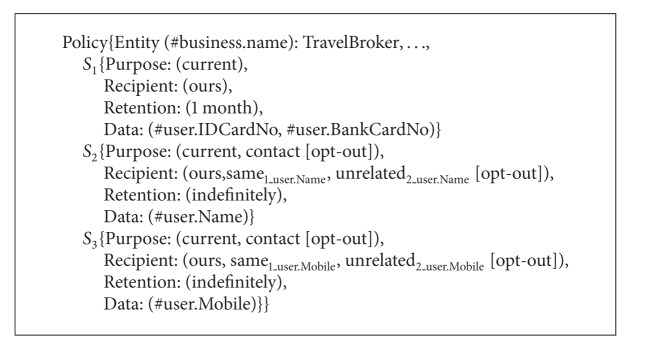
P3P privacy policy for TravelBroker.

**Table 1:** *Recipient* values of *Data* elements in Service A.

Recipient	Policy_1_	Policy_2_	Policy_3_	Policy_A_
Ours_user.name_	Including	Including	Not including	Including
Delivery_user.name_	∅	∅	∅	∅
Same_user.name_	∅	∅	∅	∅
Other-recipient_user.name_	∅	∅	∅	∅
Unrelated_user.name_	∅	∅	∅	∅
Public_user.name_	Not including	Not including	Not including	Not

Ours_user.home-info_	Not including	Including	Including	Including
Delivery_user.home-info_	∅	∅	∅	∅
Same_user.home-info_	∅	∅	Same_3_user.home-info_	Same_3_user.home-info_
Other-recipient_user.home-info_	∅	∅	∅	∅
Unrelated_user.home-info_	∅	Unrelated_2_user.home-info_	∅	Unrelated_2_user.home-info_
Public_user.home-info_	Not including	Not including	Not including	Not

**Table 2:** *Retention* values of *Data* elements in member services.

Retention	Policy_1_	Policy_2_	Policy_3_
*T*_user.name_	∞	20 days	Null
*T*_user.home-info_	Null	10 days	∞

**Table 3:** *Optional* values of *Data* elements in Service A.

Data	Policy_1_	Policy_2_	Policy_3_	Policy_A_
User.name	No	Yes	Null	No
User.home-info	Null	No	No	No

**Table 4:** *Required* values of *Purpose* values in Service A.

Purpose	Policy_1_	Policy_2_	Policy_3_	Policy_A_
Current	Always	Always	Null	Always
Telemarketing	Null	Always	Always	Always

**Table 5:** *Required* values of *Recipient* values in Service A.

Recipient	Policy_1_	Policy_2_	Policy_3_	Policy_A_
Same_3_user.home-info_	Null	Null	Always	Always
Unrelated_2_user.home-info_	Null	Opt-out	Null	Opt-out

**Table 6:** *Recipient* values of *Data* elements in TravelBroker.

Recipient	Policy_1_	Policy_2_	Policy_3_	Policy_CS_
Ours_user.IDCardNo_	Including	Including	Not including	Including
Delivery_user.IDCardNo_	∅	∅	∅	∅
Same_user.IDCardNo_	∅	∅	∅	∅
Other-recipient_user.IDCardNo_	∅	∅	∅	∅
Unrelated_user.IDCardNo_	∅	∅	∅	∅
Public_user.IDCardNo_	Not including	Not including	Not including	Not

Ours_user.Name_	Including	Including	Including	Including
Delivery_user.Name_	∅	∅	∅	∅
Same_user.Name_	Same_1_user.Name_	∅	∅	Same_1_user.Name_
Other-recipient_user.Name_	∅	∅	∅	∅
Unrelated_user.Name_	∅	Unrelated_2_user.Name_	∅	Unrelated_2_user.Name_
Public_user.Name_	Not including	Not including	Not including	Not

Ours_user.Mobile_	Including	Including	Including	Including
Delivery_user.Mobile_	∅	∅	∅	∅
Same_user.Mobile_	Same_1_user.Mobile_	∅	∅	Same_1_user.Mobile_
Other-recipient_user.Mobile_	∅	∅	∅	∅
Unrelated_user.Mobile_	∅	Unrelated_2_user.Mobile_	∅	Unrelated_2_user.Mobile_
Public_user.Mobile_	Not including	Not including	Not including	Not

Ours_user.BankCardNo_	Not including	Not including	Including	Including
Delivery_user.BankCardNo_	∅	∅	∅	∅
Same_user.BankCardNo_	∅	∅	∅	∅
Other-recipient_user.BankCardNo_	∅	∅	∅	∅
Unrelated_user.BankCardNo_	∅	∅	∅	∅
Public_user.BankCardNo_	Not including	Not including	Not including	Not

**Table 7:** *Retention* values of *Data* elements in FlightBooking, HotelReservation, and Payment services.

Retention	Policy_1_	Policy_2_	Policy_3_
*T*_user.IDCardNo_	1 month	20 days	Null
*T*_user.Name_	1 month	∞	2 months
*T*_user.Mobile_	1 month	∞	2 months
*T*_user.BankCardNo_	Null	Null	1 month

**Table 8:** *Optional* values of *Data* elements in TravelBroker.

Data	Policy_1_	Policy_2_	Policy_3_	Policy_CS_
User.IDCardNo	No	No	Null	No
User.Name	No	No	Yes	No
User.Mobile	No	No	No	No
User.BankCardNo	Null	Null	No	No

**Table 9:** *Required* values of *Purpose* values in TravelBroker.

Purpose	Policy_1_	Policy_2_	Policy_3_	Policy_CS_
Current	Always	Always	Always	Always
Contact	Opt-out	Opt-in	Null	Opt-out

**Table 10:** *Required* values of *Recipient* values in TravelBroker.

Recipient	Policy_1_	Policy_2_	Policy_3_	Policy_CS_
Same_1_user.Name_	Always	Null	Null	Always
Unrelated_2_user.Name_	Null	Opt-out	Null	Opt-out
Same_1_user.Mobile_	Always	Null	Null	Always
Unrelated_2_user.Mobile_	Null	Opt-out	Null	Opt-out
